# Novel insights into the genetics, morphology, distribution and hosts of the global fish parasitic digenean *Proctoeces maculatus* (Looss, 1901) (Digenea: Fellodistomidae) – CORRIGENDUM

**DOI:** 10.1017/S0031182024000350

**Published:** 2024-04

**Authors:** Anja Vermaak, Olena Kudlai, Russell Q-Y. Yong, Nico J. Smit

**Affiliations:** 1 Water Research Group, Unit for Environmental Sciences and Management, North-West University, 2520, Potchefstroom, South Africa; 2 Institute of Ecology, Nature Research Centre, 08412, Vilnius, Lithuania

The original version of Table 2 (page 4) in the above article contains several misattribution errors. The corrected table is presented below, with the corrected references given in bold type. In addition, authorities have been added in the first column and GenBank accession numbers are provided for novel sequence data identified in this study. All references in the table appear in the original article. The authors apologise for the errors and omissions and wish to correct them through this notice.

**Table 2.** Sequences used for phylogenetic analyses of the 18S, 28S and COI gene/regions

Species and Authorities	Host	Locality	GenBank accession numbers			
			18S	28S	COI	Reference
*Proctoeces choerodoni* **Wee, Cribb, Bray & Cutmore, 2017**	*Choerodon cyanodus*	Heron Island, AUS	KX671310	KX671299	KY073877	Wee *et al*. (2017)
*Proctoeces humboldti* **George-Nascimento & Quiroga, 1983**	*Semicossyphus darwini*	Chile	MF414438	–	–	Ñacari *et al*. (2018)
	*Sicyases sanguineus*	Chile	–	KY432601	KY432628 KU236023^1^	Oliva *et al*. (2018)
*Proctoeces insolitus* **(Nicoll, 1915)**	*Acanthopagrus australis*	Queensland, AUS	KX671312	KX671300	KY073873	Wee *et al*. (2017)
*Proctoeces* cf. *lintoni*	*Fissurella costata*^†^	Chile	EU423050^2^	–	–	**Valdivia *et al*.** (**2010)**
*Proctoeces maculatus* **(Looss, 1901)**	*Sparodon durbanensis*	TNP, SA	–	**OR724714**	**OR723765** **OR723766**	Present study
	*Clinus superciliosus*	TNP, SA	–	**OR724715**	–	Present study
	*C*. *superciliosus*	Chintsa East, SA	–	**OR724716**	–	Present study
	*Diplodus capensis*	TNP, SA	**OR724708**	**OR724713**	**OR723767** **OR723768** **OR723769**	Present study
	*D*. *capensis*	DHNR, SA	–	**OR724718**	–	Present study
	*D*. *capensis*	Chintsa East, SA	–	**OR724717**	**OR723770**	Present study
	*Archosargus probatocephalus*	Mississippi, USA	AY222161	AY222284	–	Olson *et al*. (2003)
	*Sabella pavonina*^†^	Tunisia	KX671315	–	–	Wee *et al*. (2017)
	*Sparus aurata*	Tunisia	–	KX671302	–	Wee *et al*. (2017)
	*Lithognathus mormyrus*	Tunisia	–	KU052937	–	Antar & Gargouri (2016)
	*S*. *pavonina*^†^	Tunisia	–	KU052941	–	Antar & Gargouri (2016)
*Proctoeces major* **Yamaguti, 1934**	*Thalassoma jansenii*	Queensland, AUS	KX671325	–	–	Wee *et al*. (2017)
	*Monodactylus argenteus*	Queensland, AUS	–	KX671309	–	Wee *et al*. (2017)
	*Chrysophrys auratus*	Queensland, AUS	–	–	KY073875	Wee *et al*. (2017)
*Proctoeces* cf. *major*	*Octopus sinensis*^†^	Japan	–	LC618023	–	Izumi *et al*. (2021)
*Proctoeces sicyases* **Oliva, Valdivia, Cárdena, Muñoz, Escribano & George-Nascimento, 2018**	*S*. *sanguineus*	Chile	JX306110^3^ KY432595^3^	KY432618^3^ KT865207^3^	–	**Oliva *et al*.** (**2018)**
	*Perumytilus purpuratus*^†^	Chile	JQ782525^3^	–	–	**Muñoz *et al*.** (**2013)**
**Outgroup**						
*Coomera brayi* **Dove & Cribb, 1995**	*M*. *argenteus*	Hope Island, AUS	AJ224469	–	–	Hall *et al*. (1999)
	*M*. *argenteus*	Moreton Bay, AUS	–	MZ687078	–	Cribb *et al*. (2021)
*Fellodistomum fellis* **(Olsson, 1868)**	*Anarhichas lupus*	North Sea, UK	Z12601	AY222282	–	Olson *et al*. (2003)
*Gymnophallus choledochus* **Odhner, 1900**	*Cerastoderma edule*^†^	Unspecified	–	–	KF880498	Feis *et al*. (2015)

Abbreviations: AUS (Australia); TNP (Tsitsikamma section of the Garden Route National Park); SA (South Africa); UK (United Kingdom); USA (United States of America). ^1^Listed on GenBank as *Proctoeces* cf. *lintoni*; ^2^Listed on GenBank as *Proctoeces lintoni*; ^3^Listed on GenBank as *Proctoeces* sp.; †Host is not a fish.
